# The Effects of Arts, Trades, and Professions, &c. On Health and Longevity

**Published:** 1833-01-01

**Authors:** 


### The Effects of Arts, Trades, and Professions, &c. on Health and Longevity. By *C. Turner Thackrah*, Esq. Second Edition, 1832.

The force of events has drawn the attention of all men to the condition of the working classes of this empire. Forming its numerical and actual strength, giving it by their mechanical dexterity its commercial power, the stranger might imagine that our millions of agriculturists and artisans were tolerably prosperous and comparatively happy. A little experience corrects his mistake, a little observation shews how widely remote from comfort is the life of the mechanic. Born to labour, he is doomed to the factory almost before he is begotten, and the squalid urchin picking flax or cotton is the type of the future man. Those best acquainted with our great manufacturing districts can appreciate, and they only can properly appreciate, the amount of human misery, and the sacrifice of human life, offered up to the Moloch of gain. A peasantry, their country's pride, exist but in the poet's song, or are represented by the gaunt yet abject being, whom the open day sees crawling from the plough-tail to the poor-house.

The sickly sentimentality which could not tolerate a close consideration of the vices and wretchedness of the poor is chased away by the necessity of the case, and the question of relief becomes more urgent, and yet more difficult, in each succeeding year. There is a canker in the very core of England, and human wisdom does not know how deeply it may eat. There never was a state of society resembling that which exists at present in this happy land; there never were at once so much affluence and so much poverty—such a contrast of luxury and starvation—of ease and gaiety and utter hopeless misery. What the remedy for this unnatural condition of things may be, it is not for us to inquire; but, unfortunately, it falls to the part of the medical practitioner to witness too much of this moral and physical degradation of the labouring classes.

Mr. Thackrah deserves great praise for the manner in which he has drawn attention to their sufferings, for the feeling with which he has portrayed their unhappy destinies. Whilst philanthropy is esteemed honourable, his work on the Diseases of Artisans will redound to the credit of his head and his heart. We noticed the first edition of his work in a former number of this Journal, and it merely remains for us to cull a little of the new matter by which it is now enriched. The present edition contains the investigation of 120 employments, which previously were not examined. This is a more solid recommendation than all second editions now-a-days can boast of. We shall select some of these employments for notice. We shall begin with that poetic archetype of innocence, happiness, and health—the husbandman.

"HUSBANDMEN stand at the head of this division. Spending the day in the open air of the country, and in labour varied and good, they are well known to be generally healthy. Though exposed in many parts of their employment to the vicissitudes of the weather, they seldom suffer serious injury. The dyspeptic and nervous disorders, and the long train of chronic maladies so frequent in towns, are almost unknown in the country. But on the other hand, Epidemic Fevers, Cholera, Diarrhœa, and Dysentery, are certainly more severe, and I think more frequent among agricultural peasants. The purity of the air I conceive to produce both these states,—to subject the husbandman to dangerous epidemics, as well as to exempt him from chronic diseases. Epidemics, it will be remembered, depend on a *natural* change in the constitution of the atmosphere, —some alteration, possibly, in its chemical elements, some alteration probably in its electrical state, some addition most probably of terrestrial exhalations. This baneful change or addition, whatever may be its precise character, is of course a *natural* change or addition, and will be most felt where the atmosphere is most natural. The Epidemic miasm, travelling on the wind, sweeps over the country without obstruction, but is checked by towns and crowded population. For here the atmosphere is so largely impregnated with animal effluvia, smoke, the dust and gases of manufactures and arts, that it can be but partially affected by the addition from without; and the townsman consequently inhales only a diluted miasm. So far he has the advantage of the countryman; but shortly we shall have to turn the scale by reference to the numerous and distressing civic disorders from which the husbandman is exempt. The sporadic diseases chiefly found in agricultural districts are inflammation of the lungs, pleurisy, and rheumatism, or rather those painful affections of muscles to which the term rheumatism is popularly applied.* With the exception of the labourer's debauch at a fair or feast, or the master's taking too much after market, husbandmen are generally temperate. I scarcely need add that longevity is more marked in this

* "*M. Patissier*, in his Traitè des Maladies des Artisans, states Husbandmen to be 'tres-sujets aux pleurisies, aux pèripneumonies, à l'asthme, aux coliques, aux erysipèles, aux ophthalmies, aux esquinancies, et aux rheumatismes, qui reconnaissent pour cause occasionelle l'air et la mauvaise nourriture.'"

than in any other class of society. Before concluding the subject, I must notice the inhabitants of Fens. Here ague is well known to prevail; but of late years, draining has greatly diminished the frequency of this malady. In one district of Lincolnshire, where navigable canals have vastly reduced the swamps and ditches, we were informed that the cases of ague are not more than one-tenth of their number twenty years ago. Chronic diseases of the spleen and liver are occasionally met with as effects of ague, or of the causes which produce it.* In the marshy districts, Pulmonary Consumption is extremely rare. A general practitioner at Swineshead has seen only two cases in sixteen years. Instances of longevity are frequent. In one village in the neighbourhood of the Fens, and containing not more than 120 inhabitants, there are now eight persons nearly 90 years of age." 10.

We fear that this picture of a husbandman, or, to use the more modern phraseology, of an agricultural labourer, is too favourable for many parts of England. Gibbon Wakefield's portrait of the poaching, stack-burning, calveless, abject wretch, is nearer the mark, in the southern counties. There can be no doubt that, with a moderate supply of healthy aliment, the occupation of the husbandman must be among the healthiest, if not actually the healthiest of human toils. It is curious that epidemics should exert their influence more ferociously in the quiet salubrious village, than in the densely peopled unwholesome city. We suspect that Mr. Thackrah is not right in the cause which he assigns for this, and indeed we are far from convinced that the law is so general as he imagines. When a village is invaded by an epidemic more severely than a town, it it probable that some peculiar predisposing or assisting causes are in operation in the former, rather than that the actual purity of an atmosphere should render it pernicious. At all events, it would require many facts to obtain implicit assent to the enunciation of such a law.

### Brass-founders.

"They suffer from the inhalation of the volatalized metal. In the founding of *yellow* brass in particular, the evolution of oxide of zinc is very great. It immediately affects respiration; it less directly affects the digestive organs. The men suffer from difficulty of breathing, cough, pain at the stomach, and sometimes morning vomiting. The brass-melters of Birmingham state their liability also to an intermittent fever, which they term the brass-ague, and which attacks them from once a month to once a year, and leaves them in a state of great debility. As a preventive they are in the habit of taking emetics. They are often intemperate. In Leeds we did not find one brass-founder more than 40 years of age; though we have since been informed that there are two brass-founders in the neighbourhood, of the ages of 60 and 70, who have continued at the employ from boyhood.† The *Turners, Filers,* and *Dressers of Brass,* if confined to this metal, do not seem to be more unhealthy than the generality of our townsmen. We observe among the filers, the hair of the head changed to green. This I suppose to result from the oil of the hair's combining with the copper in the brass particles." 102.

---

* In one case the spleen after death was found to weigh 3lbs."

† "*Aretæus,* in his chapter Περι Ασθματος, characterised, as most of his writings are, by a vivid picture of the disease, refers to the workers in brass, iron, &c., and to the tenders of the bath-fires. He considers them, it appears, affected with a moderate but durable asthma, the prelude of death—αμβολη ϑανατȣ."

In his remarks upon weavers, Mr. Thackrah observes, that the employment is unhealthy,—that the weavers by hand have low wages, and are often out of employ,—and that many an industrious man, working 13 hours a day, earns no more than 10s. or 12s. a week. An account of the mortality at one of the largest woollen manufactories of Leeds has been kept for several years. It can only be considered as approaching the truth, but even so far it is interesting and valuable. From January, 1818, to December, 1827, inclusive—10 years, about 700 operatives were employed, and of these 92 died, or 1.31 per cent. per annum. We perceive that the greater number were weavers and burlers. The latter are always females, who are kept in an irksome posture, and often in rooms too small.

### Copper-plate Printers.

"These have the good muscular exercise of their brethren of the letter-press. In blue printing, a composition of white lead, Prussian blue, and turpentine is employed instead of common ink; and the hands of the workmen are often daubed with the paint from morning to night, yet no injury appears to result; no case of cholic or palsy from the poison of the lead, have we found among these workmen. Whence their exemption? An intelligent master thinks the mixing of the mineral with boiled rather than cold linseed-oil may prevent its poisonous effects. But this is scarcely probable. Does the prussiate of potass exert any antidotal effect on the oxide of lead? We gave a compound similar to that of the printer's blue to a dog, with the same fatal result as if a like quantity of oxide of lead had been given in any other form. On the whole, I am inclined to attribute the exemption from palsy to the comparative infrequency of the process; the printing with blue ink being much more rare than the printing with black. Engravers and Copper-plate printers, thought not remarkable either for temperance or excess, present few examples of old age." 44.

In considering the occupation of draymen, Mr. Thackrah observes that, though intemperate they are usually long lived. He attributes this to their early rising and being much in the open air. Draymen, however, bear injuries and operations very ill, at least such is the case at St. George's Hospital, and we believe at the other London hospitals. They would seem to be peculiarly prone to diffuse cellular inflammation and sloughing, after compound fractures or lacerated wounds. Such, at least, is the remark that we should be inclined to make, from what we have witnessed at St. George's.

Mr. Thackrah observes, that engravers are apt to suffer in their eyes. We have seen some remarkable instances of this. We have under our care a young man, who is an engraver of ornaments on the locks and steel-work of fowling pieces. It is delicate work, and the glittering of the steel must be trying to the eye. This young man has a peculiar affection of the cornea of both eyes. It is affected with a sort of honey-combed ulceration which does not pass deeply, and leaves no opacity behind it. Every now and then the ulceration will nearly disappear; when a relapse, always of an inflammatory character, supervenes. His brother, who followed the same employment, was obliged to abandon it on account of affection of the eyes. It is proper to state, that the whole family appear to suffer in one way or other from ophthalmic complaints.

### GILDERS.

It is curious to observe the baneful effects of mercury upon gilders of various descriptions. It might naturally be expected that they would prove exceedingly injurious.

1. *Gilt Button Makers.* These, "in the *casting* department, are subjected not only to great heat, but to rather severe effects from the fumes of zinc. These are giddiness, headache, sickness, reduction of the appetite, and bilious disorders. The men have the appearance of ill health ; 45 is about the average duration of life. In this, however, as well as other baneful occupations, it is difficult to determine the proportion of evil which the employ and intemperance respectively produce ; for labour that distresses is generally well paid ; high wages admit considerable intervals of rest and leisure ; and leisure, by most uneducated workmen, is spent happily only at the alehouse. In *gilding*, the temperature of the rooms is 110° to 120°. But the principal evil is the mercurial vapour. Reduction of the appetite and of sleep, trembling of the limbs, soreness of the gums, and disorder of the bowels are the common effects. At Birmingham, the women employed in this department begin their work at 10, a.m. and leave it at 5, p.m. They seldom live to full age." 111.

Nothing can be more true than the remark, that pernicious as such occupations may be, they are rendered infinitely more so by intemperance. Most *uneducated* workmen, says Mr. Thackrah, fly to the alehouse. It is to be presumed that the education of the poorer classes, now begun, may do something to remedy this evil; but whilst man continues what he is, he will seek a relief from bodily labour and bodily suffering in the gratifications of sense. It is not for these highly-paid and debauched artisans that the philanthropist grieves, but rather for the millions that, by incessant and unwholesome toil, can barely obtain a miserable subsistence.

2. *Silverers of Mirrors.* The fumes of zinc appear to act chiefly on the stomach and digestive organs, producing headache, sickness, impaired appetite, and bilious disorders. Mercury is infinitely more deleterious. The silverers of looking-glasses are exposed to its influence both by inhalation and touch. They are chiefly Italians, and few bear the employment long ; some work on alternate days—many, more constantly engaged, are absent from illness for weeks or months.

"The general effects of the art are difficult enunciation, pain and constriction at the base of the chest, emaciation, debility, tremors, and lastly salivation. The gums are often wasted and the teeth left loose in the sockets. As the fingers and hands are generally the parts first disordered, it appears that the primary impression is on the nervous system at large, and is made through the medium of the skin rather than that of the lungs. Intemperate men suffer most." 112.

Mr. Thackrah refers to some cases published by Mr. Mitchell, a very intelligent surgeon of Lamb's-Conduit-street, in the Medical and Physical Journal for Nov. 1831. We shall select one of these cases, as extremely graphic and illustrative of the subject.

"P. Nash, æt. 20, of nervous temperament, commenced silvering six months ago, the trembling came on three days after he began to work, and his mouth was sore in six days ; and he has continued to suffer, more or less, up to the present time.

14th March, 1831. The speech greatly impeded; the limbs totter when he attempts to stand or walk, which he accomplishes very slowly and with great difficulty, an infirm step, and awkward gait; he is unable to convey any substance to the mouth, in consequence of the severity of the tremors; slight subsultus tendinum, confined to the upper extremities; the tongue quivers, gums slightly tender; pulse strong, rather quick; appetite diminished; sleep disturbed; body wasted; he complains as if feeling oppressed like a load across the lower part of the chest; or as if a substance lay at the bottom of the lungs, as he expresses himself, which he conceived to have been drawn in by inspiration; the breathing was quick, accompanied with strictured feeling and cough. He was nearly thrown from a bath by the violence of the trembling; a large quantity of the water was driven, by his excessive agitation, over the sides of the bath; and if two men had not held him steadily in the water, he must have been thrown out before he was capable of remaining quiet." 113.

Sometimes the men, from their inability to direct their hands with precision, are obliged to feed like quadrupeds. The metal is oxydized; and, as an oxyde, exerts its prejudicial qualities.

3. *Water-Gilders.* These " men who coat silver or other metal with an amalgam of gold and quicksilver, are exposed to the same poison as the silverers of mirrors.* They diminish its effects, however, when employed on small work, by interposing glass between the mouth and the materials; and when engaged on larger articles, by affixing to the mouth and nose a kind of proboscis, which, hanging down, opens at a distance from the source of the mercurial fumes. Notwithstanding these contrivances and every attention paid to ventilation, the art cannot be closely pursued without the induction of serious disorder. Depression of spirits, or ' nervousness,' is succeeded by trembling, sickness, depraved taste, fetid breath, and finally salivation. Palsy also is frequent; but this, as well as the other maladies, is in most cases removed by rest and fresh air. Repeated attacks, however, destroy vigour of constitution and shorten life. Men past middle age suffer so much more than others, that scarcely any are found at the employ. Water-gilders generally work but four days a week, and for about nine hours each day.

Personal cleanliness and change of dress considerably diminish the bane of this and the preceding employment. Ventilation also, and the management of currents of air through the workshops, should be regarded as much as possible.† When tremors appear, rest, fresh air, and aperients should be promptly employed; and for salivation, I have found opium the most efficacious and speedy remedy." 114.

It is curious to observe the differences produced by different divisions of the same craft. Though severe bodily exertion would certainly appear to establish a great tendency to diseases of the heart and arterial system, yet we almost invariably find that the men who employ it are infinitely more healthy than those whose occupations are more sedentary. The following passages will prove what we have asserted.

---

* " The superintendent of a manufactory told us, that from the sweeping of the chimnies on one occasion, he collected twenty pounds of good quicksilver."

† " In the Dict. des Sciences Med. Tome XXVII. are a plate and description of a contrivance by *M. Dacier* for the removal of noxious fumes, and which obtained the prize of 3000 francs, bequeathed by *M. Ravrio.* This person, who made a fortune as a *marchand de bronze d'or,* was so impressed with the sense of injury done by the Gilder's employ, that he left the prize for the discovery of some means of prevention."

### Workers in Gold and Silver.

*Workers in Silver* "have different degrees of muscular exertion according to their departments. In the process of *casting* and *moulding*, the men have moderate exertion and generally stand. The slight dust which arises from the charcoal or brick-powder, does not produce apparent effect. In the *chasing*, *hampering*, *mounting* and *pumicing*, the workmen sit, and are in consequence considerably more affected with disorder of the digestive organs. In the hampering, where they are obliged to lean much forward, the men are decidedly paler than in the mounting, where they sit upright. In the *polishing* they have an alternation of sitting and active exertion, for while one man turns the wheel to which the brush is affixed, the other holds the article to be polished. The men appear more robust in this, than in most of the preceding departments. *Stamping*, effected by raising a great weight and then allowing it to fall, is a laborious process. Each man is supposed to lift 80lbs. 500 times a day. In most of the rooms charcoal is burnt, but its gas does not produce a sensible effect, except when the apartments are low and the roofs of the common, instead of the pottery form. The 'blue vapour' in this case affects respiration at the time, and establishes a morning expectoration of mucus. In no department are the men crowded. Workers in gold and silver earn good wages, live well, and are not generally intemperate. In the department of stamping, which occasions profuse sweating, we find the men to take each during the day about three quarts of porter, twice the quantity consumed by individuals in other departments. It does not, however, seem to be injurious. The stampers were the most healthy men in the great London house we examined. In no department did we find aged operatives; but this seems to arise rather from the preference given to young men, as more expert in the improvements of the art, than from any thing baneful in the employ. A master of 12 or 16 working-silversmiths has since informed us that he has two or three between fifty and sixty years of age, and that, on examining a club of 100 men, he found as great a proportion of aged, as town-life commonly exhibits. He makes some general remarks, which I beg to insert in his own words —'Their habits are various, say two of every dozen are rather abstemious, taking about a pint of malt liquor per day, and spirituous liquors not once a month, and live regularly; eight of the same number are men who live well the first four or five days in the week, that is, eating meat two or three times a day, and drinking perhaps from two to four pints of beer. They then appear dull and heavy, but in the last two days they 'study Abernethy,' as we say; take perhaps no meat, and water instead of beer, which makes them as cheerful as possible, aided a little by the idea of being near the eating and drinking days. The remaining two, or one at any rate, is a regular drunkard, taking from four to eight pints of beer per day, and perhaps three or four glasses of spirits in the same time. Some of this class die at 30, but others are in the workhouse, and live to 50 or 60.'" 48.

*Gold Beaters* are employed for about half the day in hammering the metal, and during the remainder, in spreading the gold leaf on paper. Thus there is an alternation of labour and comparative repose. The men enjoy good wages, are healthy and robust.

*Jewellers and Workers in Gold* are a distinct class from the Silver-workers mentioned above.

"They are subjected, not only to the evils of confinement, but to the effects of gases evolved in the manufacture. These are, the gas from the coke employed, as in collecting the gold from the sweepings of the floor; the gas from the charcoal used in melting; and the vapour which arises in the process of *dry* colouring, from the fusion of saltpetre, alum, and common salt. The last produces such distress in the head and nervous system as to make it particularly disliked by the men. *Wet* colouring, in which mineral acids are used, I believe, is comparatively innoxious.* The jewellers' work-rooms are generally crowded, and the atmosphere consequently fouled by respiration, animal effluvia, and the smoke of lamps, as well as by the specific exhalations of the manufacture. Its temperature is generally raised, and in summer the heat is excessive. The labour is light; but the confinement to a leaning posture, with the head much depressed, and the elbows generally fixed to the sides of the trunk, for ten, fourteen, or sixteen hours a day, is irksome and injurious. Intemperance is general, and dram drinking especially prevalent. The disorders of which jewellers principally complain, are pains and soreness of the chest, disorders of the stomach and liver, and plethoric affections of the head. They enter the employ about 13 or 14 years of age, and are obliged to abandon it generally at 45-50. In an establishment of 37 men, two were under 20 years of age, twelve were between 20 and 30, thirteen between 30 and 40, and nine between 40 and 50; one only had passed the age of 50. An old jeweller is worthless to the art, and seldom indeed to be found. A master observes, that 'the men drop off from work unperceived and disregarded. I am quite at a loss to know what becomes of them. When they leave off working, they go, and are seen no more. Some, perhaps, become applicants for charities; but so few have I known of the ages of 60 or 70, that leaving work, they seem to leave the world as well, a solitary one appearing at intervals to claim some trifling pension, or seek admission to an alms' house.'

This is a melancholy, but, I fear, a correct representation of the end of artizans in other manufactures, as well as this, where health is either forgotten, or deliberately sacrificed to lucre, and where this lucre is devoted to intemperance —where the high wages moreover of a baneful employ, afford opportunities of absence from work for hours, or days—and where this absence or interval, instead of being devoted to the refreshment and renovation of the animal frame, harassed and injured by labour, is wickedly perverted to the induction of effects, more baneful than those of any art or occupation." 116.

It is more than probable that a life of active or severe bodily exertion, even when conjoined with temperance, is not so conducive to longevity as a life of ease. Bodily labour appears to induce, with remarkable constancy, a disposition to hypertrophy of the left ventricle of the heart, and alterations of the coats of the arteries. It is not surprising that it should do so, for the circulating system being put to great efforts, is naturally worn out the sooner. But although such a mode of existence might not conduce to absolute longevity, there can be little question that it would allow a fair share of life, and would ensure, until the age of 40 or 50, a robust frame and a

---

* "*Merat* speaks of the *derochage* and the consequent inhalation of gas from the acids as injurious. 'Beaucoup trouvent la le germ de diverses maladies de poitrine, de la Phthisie meme, ou au moins d'un etat languissant presque continuel.' '—le teint pale et plombe.' I doubt the production of consumption or other organic disease of the lungs by the inhalation of the gases. Most of these act chiefly on the nervous system, and affect the lungs, only by depressing this system and thus reducing the general health. I can readily suppose a state of great languor and defective carbonization of the blood, induced by the constant respiration of an atmosphere largely impregnated with gas from a mineral acid; but in this country workmen are not *constantly* exposed to such annoyance.

strong constitution. We should then possess a sturdy population, and our mechanics would be the raw material of excellent troops and hardy sailors. But this is an ideal picture. Labour there is, but such is society, and such is man, that whilst its injurious effects are developed, its beneficial ones are smothered and destroyed. The artisan, whose ingenuity, or the baneful nature of whose craft procures him high wages, flies to the stimulus of gin —the squalid wretch who labours for 13 hours in the 24 to obtain a pittance of ten shillings per week, has to fight against famine and filth, perhaps in addition to gin. This is probably more numerous than the former class, but whether it be or be not, our manufacturing population is such as we see it in Mr. Thackrah's pages,—on the whole, a wretched and depraved one.

We have glanced at the workers in mercury, silver, and gold. A more dismal scroll of evils from the agency of lead unfolds itself.

"*The Manufacturers of White Lead* are subjected to its poison, both by the lungs and the skin. The dust and exhalation are most from the *white-beds* and the *packing*; little from *smelting*. There is only stench from the *grinding*, and neither dust nor smell from the *blue-beds*. Such at least was the statement of the managers of an establishment at Hull; for we were not permitted personally to inspect the process, though we examined the men. In several departments the heat is such as to produce sweating. Drinking, however, is less than in many other hot employments, and white-lead preparers are not as a body intemperate. In all departments the men and women are sallow and thin, and complain frequently of head-ache and loss of appetite. The effects of the lead are most marked in the white-beds and packing departments. Here, men soon complain of head-ache, drowsiness, sickness, vomiting, griping, obstinate constipation, and to these succeed colic or inflammation of the bowels, disorders of the urinary organs, and, finally, the most marked of the diseases from lead, palsy. We observed the muscles of the fore-arm more frequently and sooner to suffer than other parts. The eyes are also affected with chronic inflammation, or reduced nervous power. Persons commence the manufacture about the age of 20; many soon leave from broken health; those who endure the employ do not remain on the average longer than the age of 45, and during one-third of these 25 years, the men are laid up in bed, or decrepid from colic or palsy. The oldest man known in a large establishment at Hull, we found to have attained the age of 54. But he is now unable to work. It is 16 years since he entered the employ, and during this period he has been laid up 28 times from serious disease! Each attack has been worse than its predecessor. He has been on one occasion 19 weeks in bed, with scarcely the power of stirring a limb, and was a month without any evacuation from the bowels. This miserable man is now partially paralytic; he has scarcely any motion in either wrist, and his lower extremities are so weakened that he can scarcely trail himself along even with the aid of a crutch. His haggard countenance and emaciated frame give the appearance of the age of 80 rather than of 54.

No person can be a month in the worst department without a serious attack of disease. Drunkards suffer most. One of them was said to have been suddenly seized with violent insanity while packing lead, and to have died soon after. Persons do not work in the lead manufactory more than five days a week on the average; and as no man could be induced to remain in the destructive departments, there is a regular change of duties. Thus, though none are destroyed, all are exposed in turn to the most baneful process.

What means can be used to improve the state of these wretched operatives? Last year I examined with care the agency of white lead, which was said to have been rendered innoxious by a peculiar process. I regret to add, that I

cannot support the statement of the projector. Will any chemical process avail to prevent the poisonous effects of this mineral? Can any substitute be found for its use in our arts and manufactures? For paint, Mr. Parkes, the chemist, recommends carbonate or oxide of zinc, which, if not wholly harmless, is a less noxious substance, and states that, though not quite so white, it keeps its hue longer than the common carbonate of lead. One means, at least, of prevention is quite practicable—cleanliness. The success of this simple measure at one manufactory,* warrants our belief that more than half the diseases of lead preparers would be prevented by washing and brushing the hands and skin whenever they leave work, cleaning the mouth, changing the dress, and the regular use of the bath. A linen dress is also recommended as excluding from the skin much of the dust which would enter through woollen. The rooms in which the process are carried on, ought of course to be spacious and well ventilated, and there should always be a strong draught through the furnace. A subsidiary chimney, anterior to the ordinary one, is mentioned by Dr. Christison as particularly efficient in carrying off the exhalations from the rakings. Men should never be allowed to take their meals in the workshops. Fatty aliments are recommended as a preservative from the poison of lead." 106.

We see the effects of high temperature in the instance of sugar refiners.

"They are exposed to more heat than almost any class of operatives. The temperature in which they work is 70°, 90°, and sometimes 120°, and that of the stoves is 150°, 180°, and often 200°. Germans bearing the work better than Englishmen, are almost exclusively employed. Though dressed only in flannel shirts and linen trowsers, they perspire profusely: on coming out of the stoves, however, they take care to rub the skin dry. A disagreeable acetous exhalation arises during the process, but does not appear to affect health. The steam also is sometimes so great as to prevent the men seeing each other. A barrel of ale placed in the sugar-house allows free potation; much indeed is taken, from three to four, or even five quarts each per day; but the men do not appear to suffer from this quantity; and drunkenness is rare. They work from three a. m. to three p. m. The labour is great. Sugar-refiners are healthy and remarkably muscular. They never suffer from the complaints commonly termed colds. They are said to be rather frequently affected with hernia, to be subjected to rheumatism, and to be worn out, or die consumptive, generally before they reach the age of 50." 135.

We believe that we have noticed the most interesting portion of the new matter introduced into this edition of Mr. Thackrah's work. It is unnecessary for us to dilate upon its merits, or to recommend professional men to make themselves acquainted with its contents. Setting aside other considerations, it must surely be worthy of a philosophic mind to investigate the

---

* "In an extensive lead factory in the vicinity of the metropolis, in which the colic peculiar to such places was formerly very prevalent, that disease has become so rare, that medical assistance has not, for some years past, been required. Many have supposed that the fumes of the lead induced the disease; but the remedy was found by tracing the cause to a more direct source. Workmen are seldom very strict in regard to cleanliness. The probability of particles of the mineral being conveyed from the hands amongst the food was suggested, and an order enforced that before any of the workmen should leave the factory to go to meals, their hands should be thoroughly washed, and that nail brushes should be used to prevent any of the lead remaining where it was most likely to adhere. The success of this plan, under strict superintendence, has been complete.—*Alcock on the Education of the General Practitioner.*"

effects of man's pleasures, pains, pursuits upon himself—to study the corporeal consequences of moral and physical impressions—to view society as it is, and not as its tinselled exterior makes it seem—to see civilization in its practical working. A philosophic mind, we say, will be improved by an inquiry so inviting, so replete with interest. How the scene displayed may affect others we do not know, but we own that the sensations excited in our own bosoms have not been those of unmingled pleasure. When we see the starving diseased artisan at his loom or his furnace, we cannot wonder at that troubled aspect which great political questions wear. But this is foreign to the matter. We again recommend the Work to the public, and thank Mr. Thackrah for the valuable instruction we have derived from it.

